# Non-Melanoma Skin Cancer in People Living With HIV: From Epidemiology to Clinical Management

**DOI:** 10.3389/fonc.2021.689789

**Published:** 2021-08-04

**Authors:** Emmanuele Venanzi Rullo, Maria Grazia Maimone, Francesco Fiorica, Manuela Ceccarelli, Claudio Guarneri, Massimiliano Berretta, Giuseppe Nunnari

**Affiliations:** ^1^Unit of Infectious Disease, Department of Clinical and Experimental Medicine, University of Messina, Messina, Italy; ^2^Department of Radiation Oncology and Nuclear Medicine, State Hospital “Mater Salutis” Azienda Unità Locale Socio Sanitaria (AULSS) 9, Legnago, Italy; ^3^Unit of Infectious Disease, Department of Clinical and Experimental Medicine, University of Catania, Catania, Italy; ^4^Unit of Dermatology, Department of Biomedical and Dental Sciences and Morphofunctional Imaging, University of Messina, Messina, Italy

**Keywords:** human immunodeficiency virus, non-melanoma skin cancer, basal cell cancer, squamous cell cancer, immunedeficiency, review (article)

## Abstract

Skin cancers represent the most common human tumors with a worldwide increasing incidence. They can be divided into melanoma and non-melanoma skin cancers (NMSCs). NMSCs include mainly squamous cell (SCC) and basal cell carcinoma (BCC) with the latest representing the 80% of the diagnosed NMSCs. The pathogenesis of NMSCs is clearly multifactorial. A growing body of literature underlies a crucial correlation between skin cancer, chronic inflammation and immunodeficiency. Intensity and duration of immunodeficiency plays an important role. In immunocompromised patients the incidence of more malignant forms or the development of multiple tumors seems to be higher than among immunocompetent patients. With regards to people living with HIV (PLWH), since the advent of combined antiretroviral therapy (cART), the incidence of non-AIDS-defining cancers (NADCs), such as NMSCs, have been increasing and now these neoplasms represent a leading cause of illness in this particular population. PLWH with NMSCs tend to be younger, to have a higher risk of local recurrence and to have an overall poorer outcome. NMSCs show an indolent clinical course if diagnosed and treated in an early stage. BCC rarely metastasizes, while SCC presents a 4% annual incidence of metastasis. Nevertheless, metastatic forms lead to poor patient outcome. NMSCs are often treated with full thickness treatments (surgical excision, Mohs micro-graphic surgery and radiotherapy) or superficial ablative techniques (such as cryotherapy, electrodesiccation and curettage). Advances in genetic landscape understanding of NMSCs have favored the establishment of novel therapeutic strategies. Concerning the therapeutic evaluation of PLWH, it’s mandatory to evaluate the risk of interactions between cART and other treatments, particularly antiblastic chemotherapy, targeted therapy and immunotherapy. Development of further treatment options for NMSCs in PLWH seems needed. We reviewed the literature after searching for clinical trials, case series, clinical cases and available databases in Embase and Pubmed. We review the incidence of NMSCs among PLWH, focusing our attention on any differences in clinicopathological features of BCC and SCC between PLWH and HIV negative persons, as well as on any differences in efficacy and safety of treatments and response to immunomodulators and finally on any differences in rates of metastatic disease and outcomes.

## Introduction

The natural history of HIV has been significantly modified by the advent of combined antiretroviral therapy (cART) that has prolonged life expectancy and reduced mortality and morbidity of people living with HIV (PLWH). Even if highly active, cART cannot cure HIV and so it is a lifelong therapy because of a hidden, even though active, reservoir (1, 2) that is able to escape the treatment. Over the past twenty years, many important factors, as increased age of PLWH and (3) coinfection with oncogenic viruses have promoted the emergence of other malignant neoplasms that collectively are classified as non-AIDS-defining cancers (NADCs) and that, over the years, overtook the incidence of AIDS-defining cancers in PLWH (4–14).

Non-melanoma skin cancers (NMSCs) include primarily basal cell (BCC) and squamous cell carcinoma (SCC). They represent the most frequent malignant neoplasms in the white population, with a worldwide increasing incidence (15). NMSCs develop from epidermal cells and their incidence increases in older age. The pathogenesis is multifactorial: chronic sun exposure is the main environmental risk factor. Other risk factors include increased longevity, genetic mutations, immunodeficiency, concurrent disease and dedicated therapy (i.e., psoriasis) (16). In immunocompromised patients, such as HIV positive patients, the incidence of more malignant form or the development of multiple tumors seems to be higher than among immunocompetent people. In PLWH these malignancies are often more aggressive compared with the general population and they need multidisciplinary assistance (17–26).

The purpose of this review is to describe the incidence of NMSCs among PLWH, focusing on any difference in clinicopathologic features of BCC and SCC between PLWH and HIV negative persons, as well as on any difference in efficacy and safety of treatments and response to immunomodulators, and finally any differences in rates of metastatic disease and outcomes.

## Materials and Methods

A systematic search of the EMBASE and Medline databases was performed to identify potentially relevant papers reporting original research on NMSCs in PLWH. This research was performed from inception to 3 March 2021, and it was restricted to humans. Clinical trials, prospective and retrospective studies, case series, case control studies and metanalysis concerning the topic of NMSCs in PLWH published in English, Spanish and Italian with available abstracts, were selected if they addressed one or more of the following topics: BCC, SCC, basal cell carcinoma, squamous cell carcinoma, HIV. The following search strings were used: “BCC OR basal cell carcinoma AND HIV”, “SCC OR squamous cell carcinoma AND HIV”. Reviews, expert opinions, book chapters and articles lacking original data were excluded. The title and abstract of all articles retrieved were check by two reviews (EVR and MGM) who selected relevant articles for full text evaluation according to predetermined criteria. Discrepancies were resulted by a third reviewer (MB). Studies were compared by title and abstracts to eliminate duplicates. A Preferred Reporting Items for Systematic Reviews and Meta-Analyses (PRISMA) flow diagram (Downloaded 03 March 2021, http://prisma-statement.org/PRISMAStatement/FlowDiagram.aspx.) was set to illustrate the review process (**Supplementary Materials**). We summarized the review according to PRISMA guidelines, represented below).

## Epidemiological Profile of NMSCs

Non-melanoma skin cancers (NMSCs) are the most frequent neoplasms in Caucasians and their incidence is increasing worldwide, with 80% diagnosed as BCC followed by SCC being both more common than melanomas (27). They are much common in white population than in skin color people. Their incidence results 18-20 times higher than that of melanoma (28). Epidemiologic studies highlight that the worldwide incidence varies widely. In fact, BCC has higher incidence in equatorial latitudes and lower in polar latitudes. Australia is the country with highest incidence of BCC, followed by the US and Europe, although the real incidence is globally underestimated (29). In Australia the rate for BCC is more than 1,000 per 100,000 person-years (2,448/100.000), followed by Europe (91 in women and 129 in men per 100,000 person-years) and the US (450 per 100,000 person-years). Cutaneous SCC is the most common skin cancer, behind BCC, and it represents approximately 20 percent of NMSCs (30). Its incidence increases more quickly with age than BCC. In PLWH cancer is becoming a growing problem representing now the first cause of death. It is clear that cancer risk is higher in PLWH in comparison with the general population (31), less clear are the reasons behind it. The advent of cART has improved the morbidity and mortality of PLWH, prolonging their life expectancy (32). A large body of literature has highlighted that HIV infection is associated to an increased risk of several different type of cancers besides NMSCs, such as lung cancer, cancer of the colon and rectum, Hodgkin disease, hepato-cellular carcinoma, head and neck SCC (HNSCC), conjunctival SCC and anogenital SCC (10, 17, 19, 20, 33, 34). BCC in PLWH show a 1.8-fold increased risk in comparison with HIV negative people (35), but it could be better in patients that have a good control of the infection. The occurrence of multiple BCC in PLWH without additional risk factors is uncommon. In HIV positive patients BCC is essentially more frequent than SCC (36) and ratios around 4:1 of BCC versus SCC have been found, similar to the general population (4:1) (33). In a retrospective cohort that studied 36821 HIV negative and 6560 HIV positive patients it has been shown an increased risk for BCC among PLWH. In fact, in this Californian cohort, the risk of developing a BCC was about twice as likely in non-Hispanic white PLWH than in the same HIV negative population. So that, it has been denoted that patients with HIV showed a meaningful tendency to develop BCC as HIV negative persons (37). Regarding HNSCCs, they are a heterogeneous group of cancers occurring in various anatomic sites, including scalp, oral cavity, lips, oropharynx, nasopharynx and larynx.

### Focus on SCC of the Scalp

SCC of the scalp represents approximately 16% of scalp cancers (38), with a mean age of 65 years at diagnosis. It has a positive correlation with advanced age.

Known risk factors for developing SCC of the scalp are older age, history of ionizing radiation chronic scarring, androgenetic alopecia, ultraviolet light exposure, actinic damage.

Immunosuppression is a crucial risk factor for all SCCs (39) that represent the most common cancer in immunosuppressed patients, with greater potential for tumor growth, cell differentiation, and aggressiveness. Furthermore, SCC may show a higher risk of metastatic disease and death in immunocompromised patients compared with immunocompetent individuals (40).

A retrospective study showed that twenty out of fifty-tree immunocompromised patients affected by cutaneous SCC of the scalp had bone invasion, that is associated with poor prognosis (41).

The aggressive behavior of SCC on the scalp in immunosuppressed patients has been described by Lang et al. (42). It is recommendable to manage scalp tumors aggressively and appropriately because they are associated with important morbidity and mortality. So that, it is essential to monitor for bone invasion, recurrence, perineural invasion and metastasis. A better knowledge of the mechanisms of recurrency could be helpful to prevent morbidity and mortality in this specific group of patients. Concerning the clinical presentation of SCC of the scalp in HIV positive patients, Ferreira CP et al. have described a case report of a sixty years-old male, white, and HIV positive in use of zidovudine, lamivudine and efavirenz, presenting tumor located in scalp, progressing with rapid growth for one year. The histopathological examination revealed a diagnosis of well differentiated SCC. Immuno-virological profile revealed CD4: 62 cells/mm³; CD8: 1,654 cells/mm³; viral load: 91,000 copies. CT brain scan revealed cerebral foci of calcification in the suprasellar region as well as in basal ganglia on the left, with a diameter of 15 mm and invasion to the skull along the interparietal suture. The patient had subsequent pneumonia that was the final cause of death. Fortunately, SCC is often diagnosed before the invasion to the skull because of its slow progression. Rarely, SCC can extend to the brain and invade, in late stages, the skull and the dura mater. When this event occurs, patients may present neurological symptoms (43). Because of the anatomical profile of the scalp region, margin excision is not always possible. Preoperative imaging is essential to define the proper extent of invasion and choose the correct treatment strategy. The treatment of SCC in advanced stages is challenging starting from the multidisciplinary surgical approach needed for a proper excision. Further studied are required for advanced disease.

## Risk Factors and Pathogenesis

Among immunocompetent light-skin color people, the development of NMSCs is favored mainly by chronic sun exposure and increasing age. There are important phenotypic characteristics, such as fair skin type, light-colored eyes, red hair, northern European origin and childhood freckling (44) that influence vulnerability to solar radiation. The frequency and intensity of sun exposure are also important.

Other environmental risk factors that contribute an increased risk for NMSCs include older age, family history of skin cancer, immunodeficiency (45), previous radiotherapy, long-term immunosuppressive treatment, genetic syndromes and chronic, mostly occupational, exposure to arsenic (46).Moreover, several observational studies have documented a correlation between use of photosensitizing molecules and increased risk for BCC (47).

### The Genetic Landscape of NMSCs

Mutations of numerous tumor suppressor genes and proto-oncogenes play a key role as drivers in BCC formation (48). In almost 90% of cases, mutations that activate the Hedgehog pathway (HH) play an established role in the development of BCC (48), while SCC is characterized by a high neoantigen burden (37). In about 50% of BCC cases, TP53 tumor-suppressor gene mutations are caused by UV radiation. TP53 encodes the P53 protein involved in maintain genomic stability by regulating the cell cycle, inducing apoptosis and activating DNA repair. Furthermore, mutations identified in PTCH1 and TP53 are so-called UV signature mutations, because in most cases they are consistent with ultraviolet radiation-induced mutagenesis.

Among genetic syndromes that may increase the risk for the development of BCC, we should keep on mind Gorlin-Goltz syndrome, also called Nevoid BCC syndrome, an autosomal dominant disease with multiple lesions of the skin, pits of the palm and developmental defects (49).

Moreover, oculocutaneous albinisms and xeroderma pigmentosum, which are known as genetic diseases with deficiencies of the protective mechanisms against UVR, are characterized by multiple and early BCCs (50).

Concerning the genetic landscape of SCC, multiple studies have shown that genes altered by UVR exposition are TP53, CDKN2A, NOTCH1, NOTCH2 and p16 suppressor gene. Moreover, mutations in DNA repair pathways include missense mutations in ATR, PIK3CA, ERRB4 and NF1 (51). In addition, association between SCC and genetic syndromes as oculocutaneous albinism, xeroderma pigmentosum, Fanconi anemia, epidermolysis bullosa and Lynch syndrome has been found (52).

### A Brief Focus on Possible Links Between the Innate Immune System and NMSCs

A large body of studies highlights that innate immunity play a key role in NMSCs development and progression. Their role has attracted increasing attention recently. As well known, the innate immune system cells can recognize numerous exogenous ligands, such as infectious agents, through various mechanisms. The most important genetic pathway networks involve a crucial group of receptors, called toll-like receptors (TLRs) (53). They are a family of ten transmembrane glycoproteins that directly recognize a wide spectrum of pathogen-associated molecular patterns (PAMPs) and damage-associated molecular patterns (DAMPs), against which they activate the innate immune response and initiate the adaptive immune response (54).

TLRs play a crucial role in the activation of innate immunity, promoting cancer progression; therefore, their activation induces genes that encode for numerous inflammatory cytokines, such as tumor necrosis factor-α (TNF- α), INF-1, IL-6, IL-1, granulocyte-colony stimulating factor and different chemokines, including CCL2 and CXCL10 (54, 55).

It has been observed that some TLRs are involved in the pathogenesis of numerous inflammatory and autoimmune skin disorders. Particularly, there is evidence that Imiquimod, a synthetic agonist of TLR-7, presents high efficacy for treatment of superficial BCC, with a cure rate ranging from 43-94% (56).

The high efficacy of this TLR-7 agonist against superficial BCC, suggests a possible role of this receptor in the pathogenesis of BCC. As a possible consequence, polymorphisms of this receptor could change host immune responses, determining a different susceptibility to BCC and others cancers and autoimmune diseases (57).

A recent case control study performed by Russo et al. (58) highlights the possible association between the susceptibility to BCC and a functional single-nucleotide polymorphism within the promoter of TLR-7 gene (SNP rs 179008/Gln11Leu).

Further genetic research of this receptor and its ligands are needed to improve the knowledge of the pathogenesis of BCC and other UV-related skin cancers.

An increasing body of evidence shows that BCC is an immunogenic tumor (59). Several immune-related markers have been implicated in BCC pathogenesis. IL-23/Th17 related cytokines, as 17, 23, 22, play a significant role in cutaneous inflammatory diseases, but their involvement in skin carcinogenesis is controversial and is poorly investigated in BCC. A recent study of Pellegrini C et al. has highlighted the role of INF-γ in BCC pathogenesis, supporting the involvement of IL-23/Th 17 related cytokines. Particularly, it has observed that BCC is characterized by higher levels of IFN-γ, IL-17, IL-22 and IL-23. Their expression could be correlated to the severity of the inflammatory infiltrate.

Concerning cSCCs, as well known, they are characterized by high mutational burden and cellular heterogeneity (60).

The role of immunosuppression in cSCC risk is supported by higher incidence among recipients of solid organ transplants and PLWH (37, 61), suggesting that this tumor type has enhanced many elements of innate immune response compared to normal skin. The immune system plays complex roles over the entire process of cancer initiation, promotion and progression.

Presentation of tumor antigens to CD8+ cytotoxic T cells and CD4+ helper T cells by HLA class I and class II molecules, respectively, is a key component of this process. The immune response is modulated by human leukocyte antigens (HLAs), which are encoded by a cluster of highly polymorphic genes located on chromosome 6. At the same time, inflammation can facilitate cell transformation by providing pro-tumorigenic cytokines and growth factors to tumor cells and forming an immune suppressive microenvironment within the tumor, which ultimately lead to immune escape and clinical manifestation of the tumors (62). A growing body of literature shows that variation in the expression pattern of these proteins, involved in the presentation of tumor antigens to T lymphocytes, has been implicated in multiple cancers by influencing host defenses against tumorigenesis. The exact mechanisms underlying these associations need to be elucidated. The strongest association between amino acid changes and cSCC risk was found for codon 26 of HLA-DRB1. However, the true functional impact of the phenylalanine to leucine change remains to be elucidated. The identification of specific amino acid changes in the HLA class II genes, if confirmed, helps provide mechanistic clues to the relationship between HLA-mediated immune response and cSCC tumorigenesis. Future studies that examine the mechanism underlying the association between HLA class II and cSCC risk need to be performed. The immune system impacts cSCC susceptibility and pathogenesis, as evidenced by the substantially higher incidence of cSCC in immunocompromised patients. Furthermore, susceptibility to the effects of UVR is known to be genetically determined (63). Variations in immunological makeup of human hosts may influence their ability to recruit immune responses needed to prevent cSCC development. Particular HLA genetic variants are associated with cSCC in immunocompetent and immunosuppressed patients, with more evidence for class I HLA-cSCC associations in immunosuppressed patients than in immunocompetent patients. Class I HLA could play a more important role in cSCC in immunosuppressed patients because HPV may be a co-factor in tumorigenesis- class I HLA proteins present intracellular peptide antigens, including viral proteins degraded into peptides. Further researches of tumor antigens involved in cSCC pathogenesis are needed, to better understand cSCC pathogenesis from an immunological point of view, and try to provide an effective prevention and treatment of cSCC (64).

### Skin Cancer, Chronic Inflammation, and Immunodeficiency: A Mènage A Tròis

Cutaneous manifestations often may reveal themselves important clinical clues of many diseases in general, including neoplastic skin diseases, that brings the patient to the physician.

The cutaneous immune system is usually linked to defense against pathogens and external agents; it can also promote the neoplastic process and tumor progression through inflammation.

**Figure 1:**
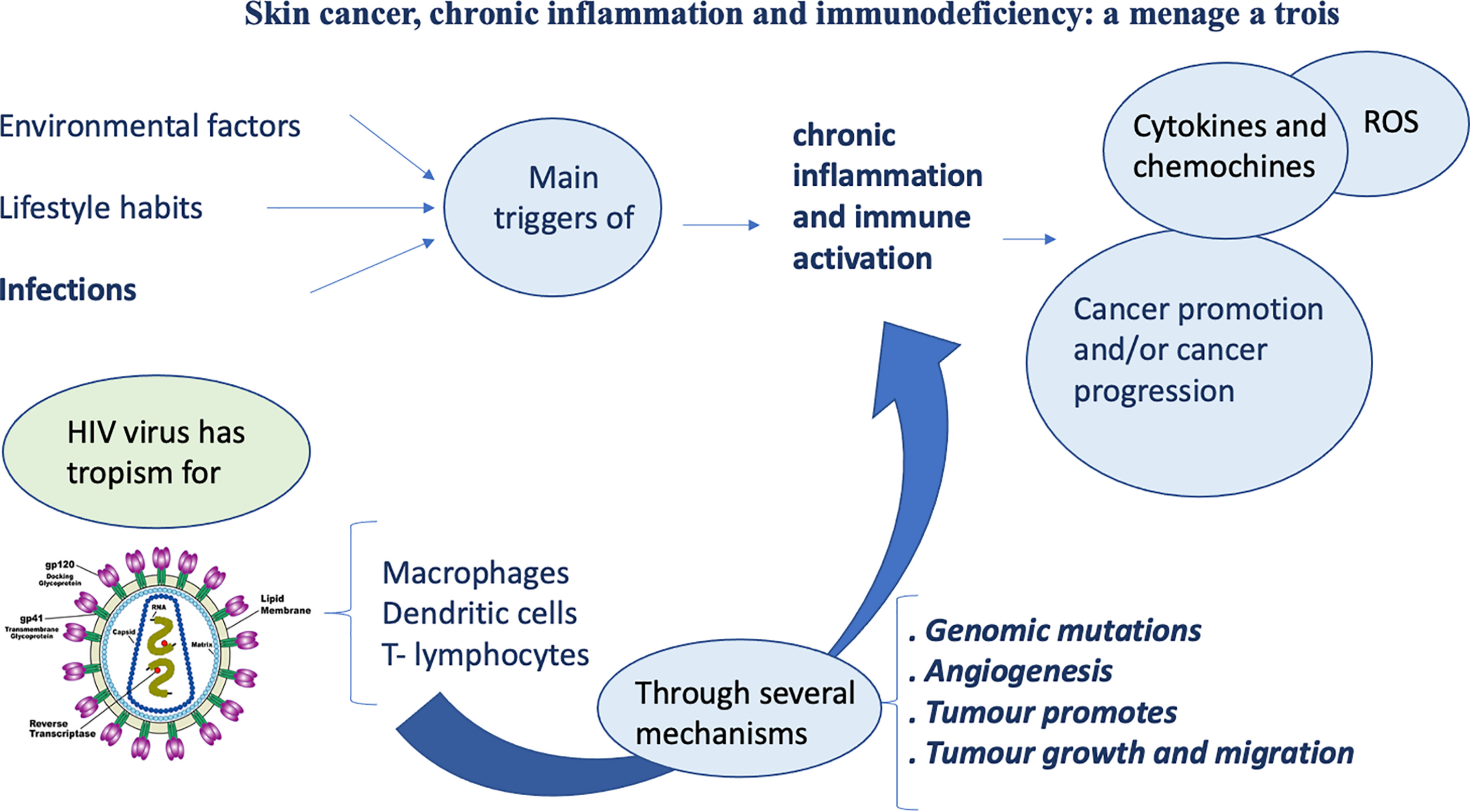
Chronic inflammation and immune activation: a delicate balance.

As known, inflammation plays a key role in oncogenesis (**Figure 1**). Different kinds of cancers arise from infections or chronic inflammation that represent the main promoters of chronic activation of immune system. This prolonged immune activation triggers various stages of carcinogenesis.

As known, immunodepression HIV-related determines an increased risk of tumors (65).

Moreover, HIV shows a tropism for cells of the human immune system, such as macrophages, dendritic cells and T-lymphocytes. HIV infection, through different processes, leads to the reduction of CD4 T-cells to a critical level. Below this level, cell-mediated immunity is lost, and this event allows the rise of opportunistic infections and AIDS development.

**Figure 2:**
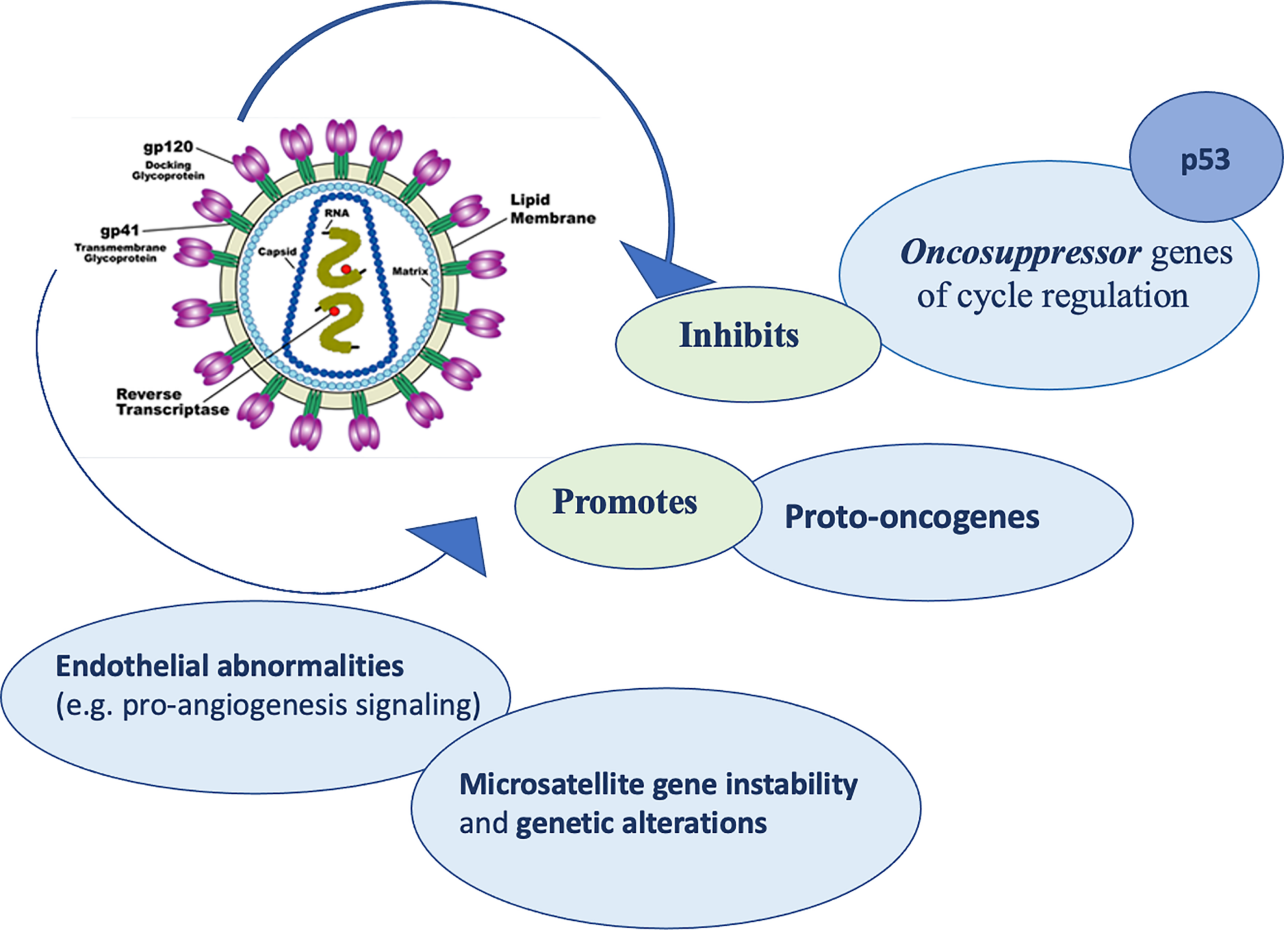
The pathogenetic role of HIV.

Regarding the mechanisms by which HIV virus induced lytic activities, Pope et al. (66) suggested that direct contact between CD4 T cells and HIV pulsed dendritic antigen-presenting cells triggers replication of the virus, leading to a death to both cell types. Furthermore, delayed-type hypersensitivity tests usually have been used as monitors for the progression of the infection, because of the compromise of cutaneous immune system is crucial (67). When CD4 and antigen-presenting cells count decrease meaningfully, skin becomes susceptible to numerous opportunistic infections and neoplastic diseases. In addition, HIV virus seems to activate proto-oncogenes (68), cause alterations in cell cycle regulation and inhibit tumor suppressor genes including p53 (69). Moreover, HIV could determine microsatellite gene instability and genetic alterations, promoting formation of different cancers, including NMSCs (70) (**Figure 2**).

Finally, HIV infection may booster pro-angiogenesis signaling that could lead to endothelial abnormalities. These alterations could promote tumor growth and metastasis (71).

Cutaneous malignancies are the majority of cancers among HIV positive patients (72) and NMSCs are now the most frequent cutaneous malignancies among PLWH. The main risk factors for NMSCs are similar to HIV negative people. Accumulate worldwide studies have shown that NMSCs are usually more aggressive in immunocompromised patients, as evidenced by an increased risk of metastatic disease and mortality in comparison with immunocompetent individuals (73). Frequent opportunistic infections represent also important risk factors for NMSCs (74).

In a study by BURGI et al. (72), cART therapy was associated with lower rates of NMSCs, whereas the standardized incidence ratio (SIR) for NMSCs was reported not to be decreased in the post-cART era among patients recorded in the Swiss cohort study (36). Moreover, a study by Silverberg et al. has suggested that the cART use is associated with decreased risk. Generally, PLWH with BCC and SCC tend to be younger, to have an increased rate of recurrence and they seem to have an overall poorer outcome (75). They often present with more advanced stages of the disease, with a greater degree of infiltrative disease and poorer outcomes (76). In PLWH possible etiologies of NMSCs include the HIV virus, coinfection with oncogenic viruses, such us hepatitis B virus (HBV) (77), hepatitis C virus (HCV) (78), human papilloma virus (HPV) and Epstein Barr virus (EBV) (79), cART agents and tobacco exposure. HPV skin infections are common but the exact correlation between HPV infection and the developing of cutaneous SCC remains still less clear (80). Multiple studies have reported indirect evidence supporting an etiologic relationship (81).

### Exploring the Link Between Viral-Immunologic Profile of HIV Positive Patients and Risk of NMSCs

**Figure 3:**
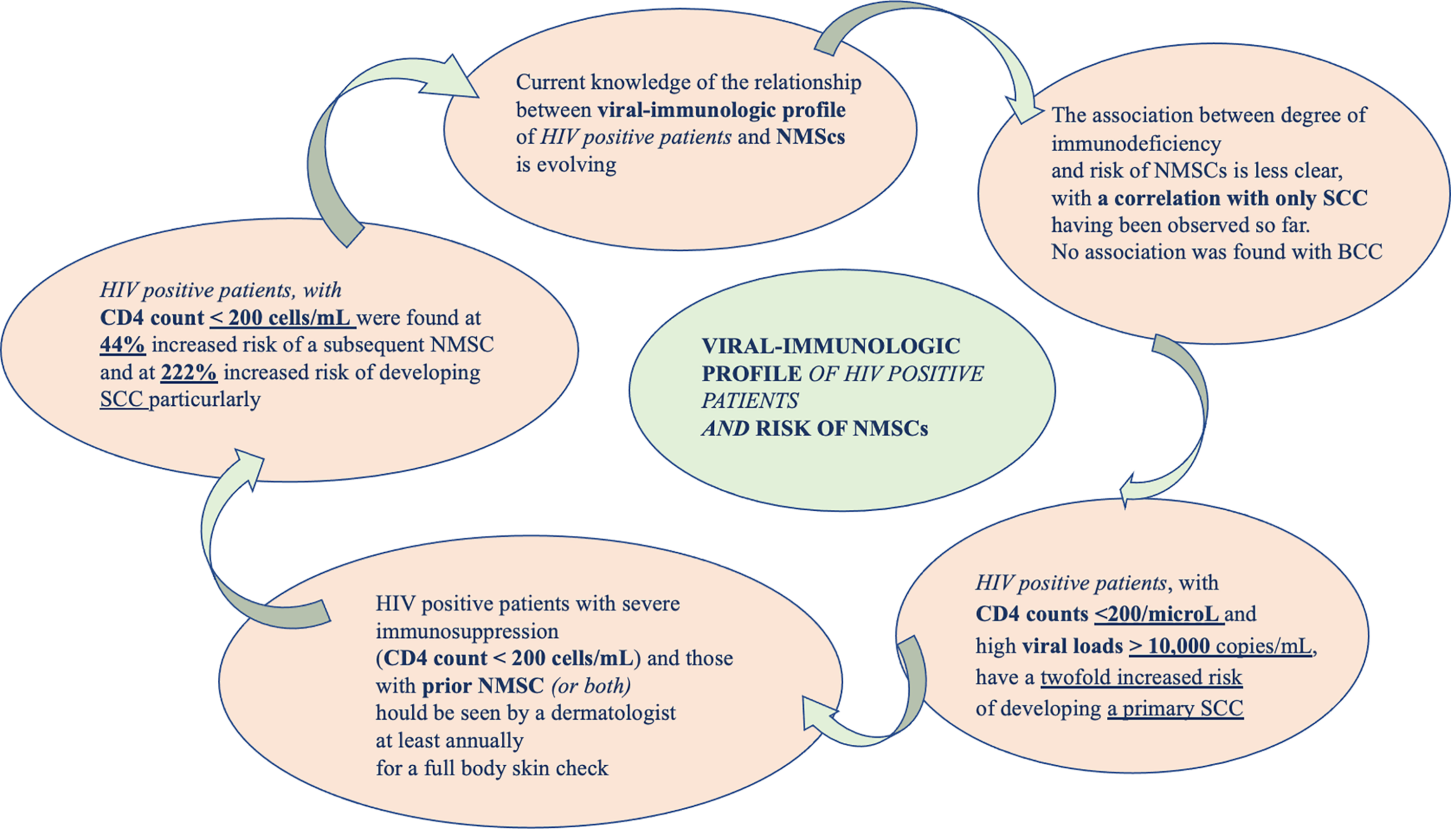
The exploration of a bond between NMSCs and immuno-viral profile of PLWH.

Current knowledge of the correlation between viral-immunologic profile of HIV positive patients and NMSCs is evolving. A peculiar correlation between decreased immune-surveillance and carcinogenic virus co-infections might favor oncogenesis, increasing the risk of developing tumors in these subjects (see **Figure 3**).

CD4 cell count is one of the main investigations in the clinical evaluation and management of HIV-infected patients and the skin is richly endowed with these cells. Immunocompetent and PLWH seemed to share the same genetic and environmental factors that lead to the formation of NMSC. Immunosuppression can increase risk to develop NMSCs, mostly SCC (82). An increased rate of neoplasms could be likely to explained by the progressive decline and dysfunction of T cells associated with HIV infection.

HIV infection causes reduced activation of both CD4 and CD8 cells and an increased synthesis of TH2 cytokine subsets. This event leads to cell-mediated immunity deficiency and accumulation of genetic mutations. HIV produces specific proteins, such as nef and tat, that alter MHC signaling and chemokine production (83).

How HIV infection could be the cause of oncogenesis it is complicated to demonstrate, especially because it seems not to be correlated with the overall immune status (CD4 counts and viral load) (84). A meta-analysis of Grulich et al. (85) have showed that immune deficiency caused an increased risk of cancer. HIV positive patients, with CD4 counts <200/microL and high viral loads > 10,000 copies/mL, have a twofold increased risk of developing a primary SCC. The association between level of immunodeficiency and risk of NMSCs is less clear, with a correlation with only SCC having been observed (37). Recently, it has been demonstrated an increased rate of NMSCs among PLWH (37). In 2017, Asgari et al. reported that non-Hispanic white PLWH had a greater risk of developing a new subsequent SCC and that this risk is correlated with lower CD4 counts and higher viral loads. The study failed to demonstrate the same for BCC. In PLWH a 15% increased risk of NMSC has been demonstrated. In particular, the possibility of a subsequent NMSC seemed to be correlated with profound immune-compromission (CD4 <200) (86).

These findings suggest that HIV-related immunodeficiency can determine an increased risk of NMSC overall and SCC in particular. In addition, the HIV viral load, often influenced by antiretroviral therapy adherence, was associated with subsequent primary SCC (hazard ratio of 2.28 with a VL above 10,000 copies/mL) but not for BCC (86). However, this study presents some limitations. The confidence intervals surrounding their HRs are not wide, suggesting that their findings were sufficiently powered. PLWH, especially those with poor immune control, could potentially benefit from targeted monitoring for SCC. In these cases, Sarah J Coates et al. recommended that patients with prior NMSC should undergo a careful dermatologic evaluation at least every year (87).

## Clinical Presentation and Diagnosis

BCC derives from the deepest cell layer of the epidermis, the basal layer of keratinocytes. Its clinical presentation is notably heterogeneous. It usually appears as a waxy, translucent, or pearly lesion that often shows a central ulceration and a raised pale border. Telangiectasias are frequent and they often bleed. Moreover, they lead to friability and poor healing. The lesion can appear atrophic and the borders can be indistinct (88). Approximately, in 9 cases out of 10, BCC arises on the head and in 7 cases out of 10 on the trunk and extremities (89). Although BCC shows minimal metastatic potential (<0,1%), local tissue effects can be destructive and disfiguring (88). Diagnosis is primarily histologically. The main histologic patterns are: nodular, superficial, morpheaform/infiltrative, basosquamous, micronodular and pigmented. Morpheaform/infiltrative, micronodular and basosquamous are considered more “aggressive growth” subtypes of BCC. Moreover, some lesions present a mixed histology.

**Figure 4:**
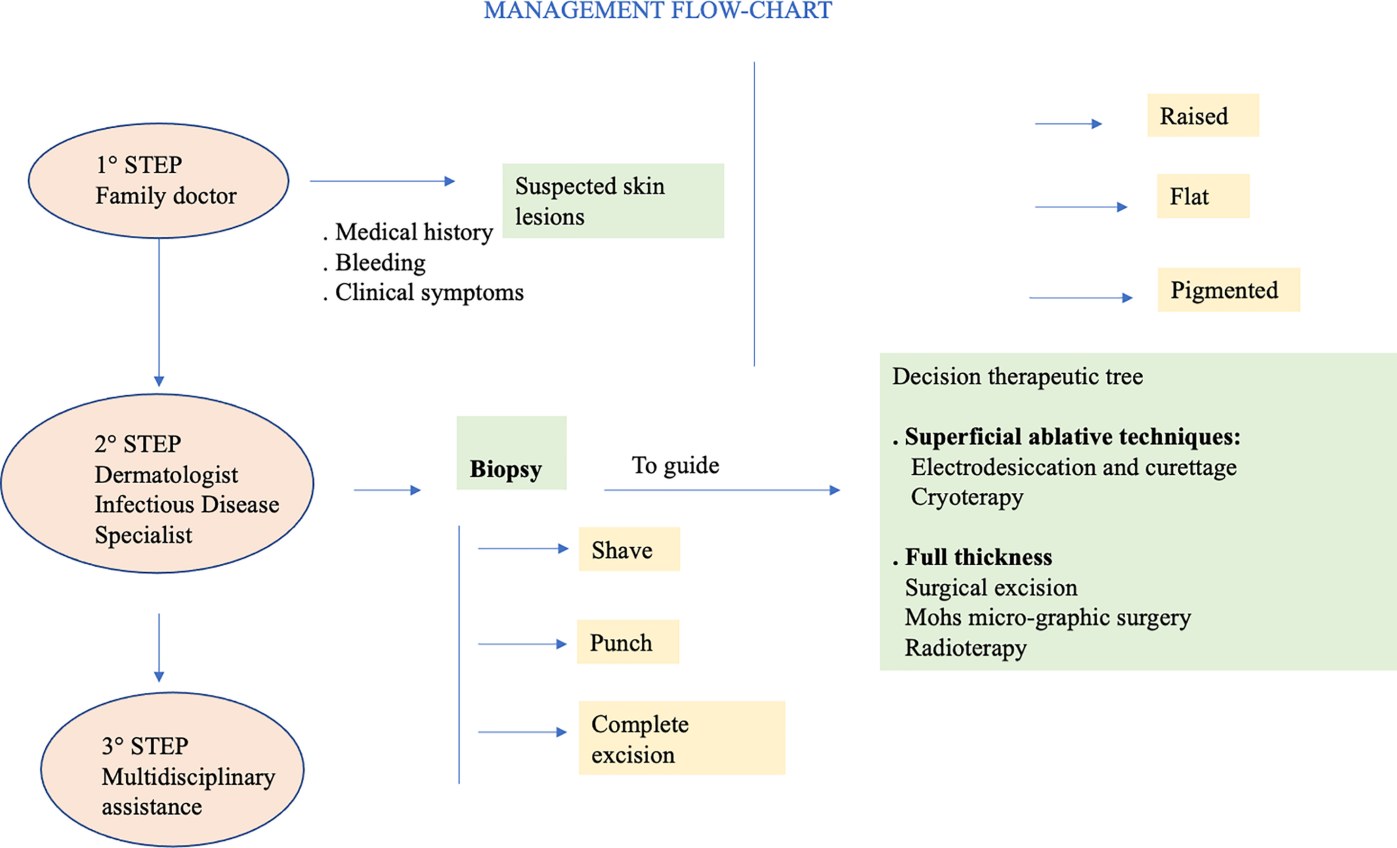
Diagnostic Management flow chart of NMSCs.

SCC arises from atypical proliferation of keratinizing cells of the epidermis or its appendages. It often develops from actinic keratosis and Bowen’s disease (SCC *in situ*) which are considered precancerous lesions. It can also grow *de novo* or on irradiated skin regions, or on chronic inflammatory skin disorders. In contrast to BCC which rarely metastasizes, SCC can metastasize initially to regional lymph-nodes and subsequently to distant regions (90). Typical clinical aspect of SCC is a raised pink papule or plaque, sometimes with scaling or an ulcerated center. The borders often are irregular and bleed easily. During the first years of follow-up, it seems to be less frequent that AKs turns into invasive SCC. When SCC arises from actinic keratosis, it appears scaly, but it tends to grow thicker, and a pink macular area develops into an erythematous raised base. Because SCC may seem quite similar to actinic keratosis, only skin biopsy accurately identifies significant cytologic atypia and invasion of SCC (89). Clinical appearance of SCC is extremely heterogeneous, and it depends also on the anatomical region and subtype. The diagnosis of SCC is primarily histologically. In all clinically suspicious lesions, a skin incisional biopsy or excision, need for a histologic confirmation, should be performed initially, depending on the size of the cancer and treatment approach (see **Figure 4**). It is possible to perform an incisional (punch or shave biopsy) or an excisional biopsy of the whole lesion. Moreover, in rare cases of uncertain diagnosis, immunohistochemical markers of differentiation, such as cytokeratin or molecular biological markers can be applied (50).

Generally, PLWH with SCC and BCC present identically to immunocompetent individuals (91). BCC generally appears on the trunk, while SCC on the head and neck regions. Superficial type BCC is the most typical clinical and histologic presentation, which tends to be multiple, involving the trunk. Generally, in PLWH malignant cancers show a more aggressive phenotype and poorer survival rates in comparison with immunocompetent persons. NADCs show often earlier age at onset, higher tumor degree, more aggressive clinical course and/or more advanced stage at presentation, highlighting the need for prompt and aggressive treatment. More aggressive clinical course has been correlated with multiple factors, such as anatomic site, size at onset, growth rate, histologic features and recurrence after treatment (92). A substantial body of evidence on metastatic SCC highlights that head and neck are primary sites; particularly the temporal and zygomatic regions seem to have a clear tendency for metastasis, maybe because of rich and direct lymphatic drain-age to the parotid gland (93).

Nguyen et all. have demonstrated that PLWH can develop rapidly growing SCC at a young age, with a high risk of local recurrence and metastasis. Management of high-risk SCC should be aggressive and not palliative in PLWH (92). However, cART has certain improved the life quality of PLWH and their outcome that appears more similar as in the general population. Several worldwide studies have highlight that in PLWH, NMSCs are usually characterized by a more aggressive clinical course, higher cancer grade, advanced stages at cancer diagnosis and shorter survival compared with HIV negative individuals (74). SCC seem to be more dangerous in the context of HIV disease. R. N. Motta et al. have described the case of a 59-year-old male patient with advanced HIV infection who presented with a highly aggressive SCC lesion scalp area with destruction of the underlying parietal bone and fulminant clinical progression (94).

Nguyen et al. (92) have described ten cases of aggressive SCC. They recorded 41 different SCC lesions: 75% in head and neck, 7% in the trunk and 8% in extremities.

based on rapid growth rate, a diameter of over 1.5 cm, a history of recurrence and/or evidence of metastasis. A total of 41 SCC lesions were recorded from 10 patients. The head and neck were the most frequently involved regions (31 lesions), followed by the trunk (7 lesions) and extremities (1 lesion). The article stated that those patients initially treated with radiation therapy and surgery combined as well as those treated with radical neck dissection had the best outcomes (92). This paper suggests that high-risk SCC should be treated aggressively and not palliatively in patients infected with HIV.

## From Prevention to Therapy

**Figure 5:**
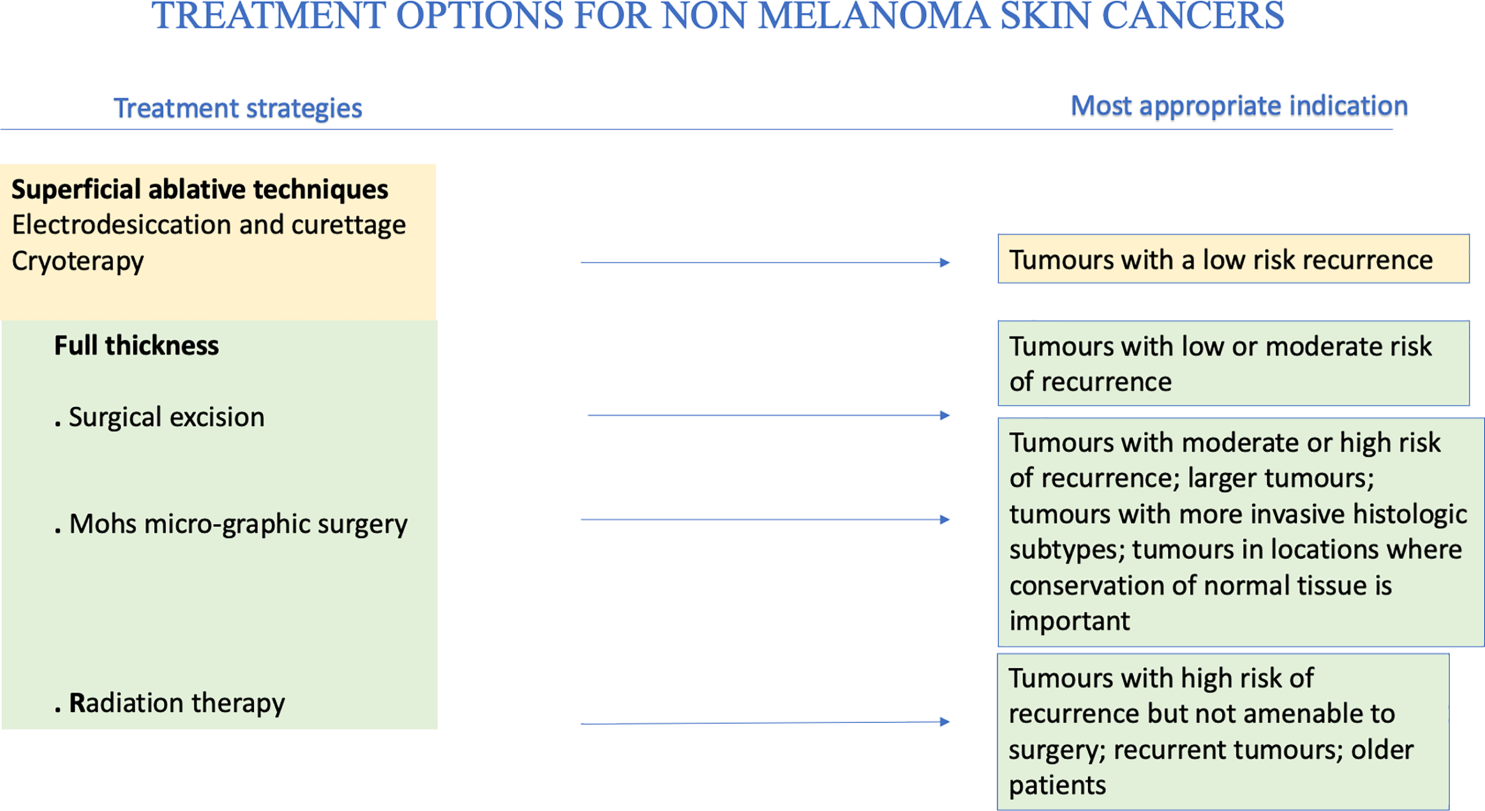
Treatment options for NMSCs.

Skin cancer can be avoided by following simple prevention rules (95). Primary prevention is of utmost importance. In particular, sun exposure should be reduced and totally avoided when at its peak during the day, and intensive tanning discouraged. Secondary prevention should be aimed to reduce morbidity and mortality, mainly through early detection of skin cancer, as close clinical evaluation of the arms, face and upper chest can uncover many lesions. In PLWH it is vital a careful evaluation with early biopsy of suspicious lesions. Precancerous lesions should be undergone an early diagnosis to prevent the development of invasive SCC. When cycle of therapy is concluded, patients should undergo a regular follow-up with evaluation of local recurrence or nodal metastasis, particularly for SCC. Other important prevention strategies include smoking cessation and prevention and/or treatment of oncogenic viruses’ coinfections, such as HPV, HBV, HCV (13, 26). HPV plays an important etiologic role in genital SCC, so that the quadrivalent HPV vaccination has been strongly suggested (96). Generally, cancer therapy is chosen on the basis of location of primary disease, extension and spread and host comorbidities. Moreover, it depends on histology, lesion aspect, size and location, as well as patient compliance. BCC and SCC should be primarily treated with complete surgical excision (97) (see **Figure 5**).

Management of BCC remains primarily surgical (98), as in immunocompetent people. Management of SCC is influenced by clinical presentation (i.e., palpable lymph nodes) and histopathologic features. Generally, a full skin examination should be performed in all patients, followed by lymph node examination and by surgical and medical management involving a team of experts. It is important to extend the excision at least 6 mm from the margins independently from the site whenever it is possible (98). Standard treatment should be applied to all PLWH with a newly diagnosed NMSCs (99); however, when combining cART with chemotherapy, potential drug-drug interactions and overlapping toxicities such as nausea and diarrhea, myelosuppression, neuropathies may occur (99). In case of overlapping toxicity occurs, it is recommendable to change cART or the chemotherapy agent rather than stopping the antiretroviral therapy or decreasing the dosage of chemotherapy (99). Many studies suggested that outcomes can be similar in PLWH with a good control of their infection and HIV negative people (100). However, PLWH with advanced disease show a poor tolerance of therapy and they more likely have worse outcomes compared with HIV negative individuals (101). In immunocompromised individual oral retinoids could be effective to reduce cancer load and to partially prevent the occurrence of new lesions. Unfortunately oral retinoids are teratogenic and that represent a limitation in their use (102). Morbidity and mortality of aggressive SCC in PLWH depend on the control of the disease in the early stages (92). People with higher risk cancers should receive loco regional adjunctive radiotherapy or chemotherapy or both and sentinel node procedures. These recommendations apply regardless of CD4 counts. There is no evidence that BCC in PLWH need more aggressive therapy. For example, Wilkins et al. recommend the use of the same treatment protocols for treatment of BCC even if there is no evidence for imiquimod for BCC in PLWH (91). Among factors influencing prognosis, any kind of immunocompromised patient present more rapid growth, an higher risk of local recurrence and metastasis, even 10 times higher (82). Intensity and duration of immunodeficiency plays a great role (103). Immunocompromised patients should be followed-up closely, at least twice a year (50). PLWH can die of a metastatic SCC, so the treatment of SCC in PLWH should never be less aggressive or prompt than the treatment of HIV negative individuals. Concerning metastatic SCC, it is important to keep in mind that late treatment of high-risk SCC could lead to metastatic diseases especially in immunocompromised people. Moreover, perineural invasion is clearly linked to recurrence and higher risk of metastasis. Generally, the most chosen surgical option in these high-risk cases is Mohs surgery. But the presence of perineural invasion requires additional adjuvant therapy (104). Similarly, high-risk SCC in HIV infected patients should be treated initially by ablative therapy with histologic control and, if necessary, adjuvant therapy. A retrospective study of Nguyen has illustrated the potential for rapid growth of SCC in HIV infected people. An initial less aggressive therapeutical approach in PLWH is linked to higher rates of recurrence, metastasis and death. For this reason PLWH with SCC should receive a combination of surgery and radiotherapy or of surgery and radical neck dissection (92). NMSCs are a striking example of immunodeficiency-related neoplasm, and they offer further opportunities for therapeutic and pathogenetic insights. In fact, multiple clinical phenomena highlight the close correlation between immunity and skin cancers.

The main therapeutic techniques, superficial ablative and full thickness, for NMSCs will be broadly reviewed above (see **Table 1**).

**Table 1:** Classification of BCC according to risk for recurrence (105, 106).

LOW RISK	INTERMEDIATE RISK	HIGH RISK
Superficial primary BCC	Superficial recurrent BCC	Clinical forms: Morpheaform or ill-defined
Nodular primary BCC when:	Nodular primary BCC when:	Nodular primary BCC when:
<1 cm in intermediate risk area	<1 cm in high-risk area	>1 cm in high-risk area
<2 cm in low-risk area	>1 cm in intermediate risk area	
	>2 cm in low-risk area	
Pinkus tumor BCC		Histological forms:
		Aggressive
		Recurrent forms

### Surgical Excision

Surgery is the treatment of choice. Depending on the affected area, it can be followed by plastic reconstruction. Moreover, histological examination of the excised tissues allows diagnosis, prognosis and treatment tailoring.

In SCC, surgical excision is immediately followed by histopathological examination of excision margins, which allows to confirm the cancer type and assess the absence of cancer cells from the resection margins. Another procedure to obtain the same result is micrographically controlled surgery (MCS). For low-risk NMSCs limited to dermis, traditional excision preferred (89). Aesthetically, excision offers better results than ablative techniques. Moreover, it offers the advantage of obtaining specimens for histologic examination. With surgery, cure rates are higher than 90%. It is neither recommended, nor cost-effective, storing frozen sections of tumor margins of every suspected NMSC. MMS is applied for recurrent tumors, tumors in high-risk areas, tumors ≥ 2 cm, recurrent tumors, tumors which margins are not clear and tumors in cosmetically sensitive areas (107). Wide removals should be done when margins are smaller than the recommended safety margins due to the tissue shrinkage, while re-excision should be done for operable cases in the event of positive margins (108). In the context of high-risk SCC, usefulness of a sentinel lymph node biopsy is still not clear (109). In fact, SCC does not invade deeper tissues as quickly as cutaneous malignant melanoma. The reason consists in absence of lymphatic drainage in superficial dermis and epidermis. Therefore, SCC is less likely to spread *via* lymphatics. There are still no guidelines about how to approach regional nodal disease in patients with SCC. Moreover, available directions are based on studies concerning head and neck mucosal SCC (110). Patients affected by metastases from SCC spread to lymph nodes should be treated surgically, as well as patients with melanoma or Merkel cell carcinoma. When surgery is not indicated, e.g., for patient-related factors, a nonsurgical approach by a multidisciplinary group should be evaluated.

### Radiotherapy

Radiotherapy (RT) may be applied in an adjuvant setting, after surgical resection, in patients with high-risk features. A host factors as immunosuppression is considered by the American Joint Committee of Cancer (AJCC) as a risk for having a poorer outcome when diagnosed with NMSC. Obviously, the presence of other risk factors such as location, particularly ears and lips, poor differentiation and perineural invasion (PNI) can worsen outcomes. American Society for Radiation Oncology (ASTRO) guidelines recommends postoperative radiotherapy (PORT) in the setting of chronic immunosuppression (111).

Bimodality therapy (surgery and PORT) is used in the context of immune suppression, especially with head and neck cutaneous SCC. As a matter of fact, it frequently presents a lower outcome than immunocompetent patients, with a significantly lower progression-free survival at 2 years (*p* = 0.002) (112). When necessary, adjuvant RT should not be delayed. It is demonstrated that exceeding a time of 6 weeks after the excision may worsen the prognosis (113). Irradiation volume must consider cancer location and risk factors, such as PNI, lymphatic and vascular invasion, to decide whether to include the first lymph node. The results of phase III TROG 05.01 trial (114) suggest no benefit in overall survival, disease free survival and locoregional relapse with the addition of weekly carboplatin to RT as adjuvant therapy.

RT is recommended as the only treatment modality in patients with NMSCs who cannot benefit from surgical resection. In fact, NMSCs can obtain an optimal local control because they are radio responsive carcinomas. Marconi et al. (115), using definitive RT, demonstrated that BCC had a 5- and 10-year local control of 96% and 94%, while for SCC 5- and 10-year control were 92% and 87%, respectively. It is important to keep in mind that in case of underlying genetic syndromes RT is discouraged because of higher radio-sensitivity in patients affected by Li-Fraumeni or Gorlin syndrome, ataxia telangiectasia. Furthermore, connective tissue disorders represent a contraindication to treatment whenever not under control (111).

### Cryotherapy

Cryotherapy represents a therapeutic option for BCC, although tissue destruction is not perfectly targeted. It is based on two consecutive 30-second freeze-thaw cycles and is particularly effective on facial lesions, with a 95% cure rate (116).

### Electrodesiccation and Curettage

Generally, these therapeutic options are considered only when assessing low-risk lesions. These techniques have a worse cosmetic yield than surgical excision, often ending in a round, hypopigmented and possibly hypertrophic scar (89). National Comprehensive cancer Network (NCCN) guidelines reported that curettage and electrodessication may be considered for small and low-risk primary SCC (117).

### Chemotherapy

Systemic chemotherapy has a meaningful role in the management of local advanced and/or metastatic NMSC. Aggressive management with polychemotherapy should be considered for difficult to treat cases. Usually, mono-chemotherapy should be considered as a first-line treatment (50). Metastatic SCCs are notably difficult to treat, representing a challenge for clinicians. Platinum based chemotherapeutic agents, such as cisplatin or carboplatin, can be considered for local advanced and metastatic SCCs not amenable for surgical excision or radiotherapy. Other chemotherapeutic drugs, such as cyclophosphamide, bleomycin, doxorubicin, methotrexate and 5-FU, may also be used alone or in combination (118). However, guidelines for the use of classic chemotherapy in NMSC are based on low-level evidence, as the trials had several limitations, such as lack of randomization and heterogeneous patient populations. Recently, it has been highlighted that patients with stage I and II lip SCC can be successfully treated with monotherapy *via* superficial temporal artery administration of bleomycin, in order to obtain a cure in 70.8% of patients (119). Currently, chemotherapy is recommended in NCCN guidelines in a combination with radiotherapy, especially in localized, high-risk SCCs for patients who cannot undergo surgery (117). Before the advent of molecular target therapies, metastatic BCC had been treated with various conventional chemotherapeutic agents. However, metastatic BCC is rare, and the available literature about the effectiveness of these treatments is mostly episodic. In a short review collecting twelve elsewhere published cases treated with platinum, five showed complete response and four showed partial response (120).

### Immunotherapy and Target Therapy of NMSCs: New Promising Neoadjuvant Therapy

Given actual evidence, targeted therapy and immunotherapy represent the frontiers in neoadjuvant therapy of NMSCs, being much more selective than traditional chemotherapy. Emerging clinical data (see **Table 2**) show that immunotherapy, particularly checkpoint inhibition, is a useful therapy option for advanced cSCC, while targeted therapy with sonic hedgehog pathway inhibitors results an effective treatment option for locally advanced or multiple BCC (121). The role of immune system has been linked to the occurrence of NMSCs by epidemiologic evidence that led to several studies about the immunology of NMSCs (122). These studies demonstrated the elevated number of neoantigens expressed by NMSCs’ cells that could represent the right target for a successful immune therapy. These kinds of observations have led to ongoing clinical trials based on novel immunotherapies of NMSCs as a neoadjuvant approach (123). By definition, a neoadjuvant approach aims to reduce the size of the tumor, before the subsequent potentially curative techniques. Immunotherapy acts by inhibiting immune checkpoints, eventually improving the activity of the immune system against the tumoral cells and reducing regulatory T cell-mediated immunosuppression. Unfortunately, these new treatment options appear quite expensive; moreover, immunotherapy can cause important and irreversible adverse effects (121). A thorough knowledge of SCC carcinogenesis is needed to develop new treatment approaches. The main immune checkpoints include CTLA-4, PD-1 and PD-L1, while sonic hedgehog pathway inhibitors include Vismodegib and Sonidegib, that we briefly describe above.

**Table 2:** Immune Checkpoint inhibitors.

Stage of disease	SCC	BCC
I	Nivolumab ± Ipilimumab(II)	N/A
II	Nivolumab ± Ipilimumab (II)	N/A
	Cemiplimab (II)	
III	Nivolumab ± Ipilimumab (II)	N/A
	Cemiplimab (II)	
IVA	Nivolumab ± Ipilimumab (II)	Nivolumab ± Ipilimumab (II)
	Avelumab (II)	
	Cemiplimab (II)	
	Pembrolizumab (II)	
IVB	Nivolumab (II)	Nivolumab ± Ipilimumab (II)
	Avelumab (II)	
	Cemiplimab (II)	
	Pembrolizumab (II)	

Immune Checkpoint inhibitors currently under investigation for the treatment of squamous cell carcinoma and basal cell carcinoma. Between brackets the phase of the study. SCC, squamous cell carcinoma; BCC, basal cell carcinoma; N/A, not applicable. Data extracted from https://clinicaltrials.gov/.

#### Anti-Programmed Cell Death Receptor-1 Immune Checkpoint Inhibitor

##### Cemiplimab

It is indicated for advanced or metastatic SCC in patients who are not amenable for surgery or radiotherapy. The phase I/II study (EMPOWER-CSCC-1) of patients with locally advanced or metastatic SCC has been the first trial that led to drug approval, producing a response rate of 47% in a cohort of 59 patients (124). Recently, this study led to the U.S. Food and Drug Administration approval of cemiplimab for locally advanced or metastatic SCC on September 28, 2018. The phase II clinical study of Cemiplimab in patients with advanced cutaneous SCC is ongoing and it is currently recruiting participants. (NCT02760498).

Another study (NCT03969004) is currently recruiting participants to study cemiplimab use in the adjuvant setting after surgery and radiation in patients with high risk of recurrence. Numerous ongoing clinical trials are studying the use of cemiplimab in patients with advanced BCC with a progression of disease while on Hedgehog pathway inhibitor therapy (125). Between them, the study (NCT03132636) is active, not recruiting. Another ongoing clinical trial is studying CTLA-4/PD-1 combinations, such as ipilimumab/nivolumab for treatment of advanced BCC. This study (NCT03521830) is currently recruiting participants with locally advanced or metastatic BCC.

##### Pembrolizumab

There are currently ongoing studies that are investigating the treatment of recurrent or metastatic cSCC (126). Between them, (NCT02964559) is an active study, not recruiting participants. It is also being evaluated in advanced SCC (NCT03284424), an active study, not recruiting for treatment of recurrent or metastatic cSCC.

##### Nivolumab

It is also being evaluated in advanced SCC: (NCT04204837) is an active study, not recruiting.

#### Anti-Programmed Cell Death Ligand-1

##### Avelumab

Several ongoing studies for advanced SCC are investigating avelumab with or without cetuximab (121). The study (NCT03944941) is currently open to enrollment. Another study, (NCT03737721) is currently recruiting participants with unresectable SCC treated with avelumab and radical radiotherapy. This study is called UNSCARRed study.

##### Atezolizumab

The study (NCT03108131) studies how cobimetinib/atezolizumab association works in treating participants with rare tumors that have spread to other places in the body (advanced) or that does not respond to treatment (refractory). This study is currently recruiting participants. Cobimetinib may block some of the enzymes involved in cell growth. So that, immunotherapy with monoclonal antibodies, such as atezolizumab, could interfere with the capability of tumor cells to grow and spread.

##### Cosibelimab

Cosibelimab is a fully human monoclonal antibody of IgG1 subtype that directly blocks its interactions with the Programmed Death-1 (PD-1) and B7.1 receptors (121). The study (NCT03212404), based on cosibelimab/atezolizumab association, is currently recruiting participants. The aim of this study is to assess the safety, tolerability and efficacy of CK-301 when administered intravenously as a single agent to subjects with recurrent or metastatic cancers.

#### Anti-Cytotoxic T-Lymphocyte-Associated Protein 4 Immune Checkpoint Inhibition

##### Ipilimumab

Emerging data showing ipilimumab use in SCC are limited to case reports. A patient with metastatic cSCC had a durable remission of both malignancies. Concerning BCC, there is an ongoing study regarding locally-advanced unresectable or metastatic BCC which investigates ipilimumab in association with nivolumab in one of the arms (NCT03521830) (127). This study is currently open to enrollment.

#### Hedgehog Pathway Inhibitors: Vismodegib and Sonidegib

Genetic and molecular studies have highlighted genetic mutations in the hedgehog signaling pathway characterize almost all BCCs. These alterations result in excessive activation leading to uncontrolled proliferation of basal cells. In addition, they determine loss of function of patched homologue 1 (PTCH1). PTCH1 blocks the signaling activity of smoothened homologue (SMO), a seven-transmembrane protein.

Vismodegib and sonidegib are two anti-tumor drugs targeting the HH pathway, called hedgehog pathway inhibitors (HPIs). Currently, there are no recommendations about when to prefer one molecule rather than the other. Moreover, these molecules have similar efficacy and tolerability, although they differ under a pharmacokinetic aspect (128). As a matter of fact, both are metabolized through cytochrome P450. Vismodegib is prevalently metabolized by CYP2C9, while sonidegib passes through CY3A4. Therefore, CYP3A4 inhibitors increases the blood concentration of sonidegib. Among them, ritonavir e saquinavir, two antiretroviral drugs. Whenever it is not possible to avoid the simultaneous use of sonidegib and strong inhibitors of CYP3A4, a dose reduction to sonidegib 200 mg every second day is recommended (129). Muscle spasms, alopecia, dysgeusia and weight loss are the most frequent side effects described in the literature. Of interest, many cases of SCC have been observed in patients treated with vismodegib for BCC therapy or single agent (BRAF) inhibitors, such as vemurafenib, for melanoma therapy (130).

All the current conventional treatments and ongoing trials are summarized in **Table 3**. Further studies are required to better understand the correct management of the drug, alternative dosing regimens and differences with the other HPIs.

**Table 3:** Conventional and New promising neoadjuvant therapies.

CONVENTIONAL THERAPY	
**SURGICAL EXCISION**	Generally adopted as **first step** for most NMSCs and it is considered a **potentially curative treatment**
**RADIOTHERAPY**	Effective non-surgical option and used in the **definitive, adjuvant and palliative settings**
**CRYOTHERAPY**	Reserved only for **low-risk lesions**
**ELECTRODESICCATION AND CURETTAGE**	Reserved only for **low-risk lesions**
**CHEMIOTHERAPY**	Topic mono-chemotherapy, e.g. with **5-fluorouracil** or **Imiquimod**, can be considered for **superficial lesions**
**NEW PROMISING NEOADJUVANT THERAPY**	
**TARGETED THERAPY**	Targeted therapy with sonic hedgehog pathway inhibitors is very effective in **locally advanced or multiple BCC**.
**-**Sonic hedgehog pathway inhibitors:	
Vismodegib and Sonidegib	
**IMMUNOTHERAPY**	Immunotherapy with immune checkpoint inhibitors appears to be promising for **advanced cutaneous SCC**. Several **ongoing clinical trials** are investigating their use. **Cemiplimab** is the only checkpoint inhibitor approved for locally advanced or metastatic cSCC.
- Anti-programmed cell death receptor-1 checkpoint inhibitor **(Anti PD-1)** - Anti-cytotoxic T-lymphocyte-associated protein 4 immune checkpoint inhibition **(Anti CTLA-4)** - Anti-programmed cell death ligand-1 **(Anti PD-L1)**	

Conventional and New promising neoadjuvant therapies. SCC, squamous cell carcinoma; cSCC, cutaneous squamous cell carcinoma; BCC, basal cell carcinoma.

### Target Therapy in PLWH

Immunotherapy has paved new paths for treatment of HIV-related cancers and, thanks to monoclonal antibodies and immunomodulatory drugs, have shown to be effective in HIV-related cancers. In particular, the effectiveness of checkpoint inhibitors targeting the PD-1/PD-L1 pathway in the treatment of many malignancies in PLWH it has been suggested by recent data, hopefully stronger evidence on this matter will follow with the inclusion of PLWH in immune-oncology studies. Recently, ASCO and the Food and Drug Association (FDA) have provided guidance to include PLWH in clinical trials on neoplastic diseases.

A recent FDA-approved sonic hedgehog (SHH) signaling pathway inhibitor, Vismodegib, can be used to treat locally advanced, metastatic and recurrent BCCs that are inoperable and cannot be treated with radiotherapy, showing promising results (131). Although this molecule seems to be a safe option for those patients that cannot undergo surgery for advanced and metastatic BCC, in high-risk patients the optimal treatment protocol is unknown. The safety of Vismodegib in PLWH and its interactions with cART are not well known. Recently, Scalvenzi et al. have described a case-report of a HIV positive patient with an inoperable ulcerative BCC of the ear.

After a specialistic evaluation also of the immune status (high CD4 T cell count) the patient started oral Vismodegib 150 mg daily. In about 6 months of therapy the patient obtained a complete resolution, after which Vismodegib was discontinued. The article reports good tolerance and no interactions between Vismodegib and the previous cART (132). Reports on SHH inhibitors in immunocompromised patients witch locally advanced or metastasizing BCC are rare (133). Effectual use of Vismodegib and the lack of drug to drug interaction with cART (tenofovir/emtricitabine/rilpivirine) has been described in a single case (132). Recently, Hoffmann V. et al. have described a successful case of treatment with Sonidegib in a patient on cART. However, it’s mandatory to evaluate the risk of interactions between cART and antiblastic chemotherapy, target therapy and immunotherapy (134). In fact, it is true that the new antiretroviral drugs (135) are less toxic but they still have long-term side effects that need to be careful evaluated (136).

## Conclusions and Future Perspectives

PLWH have an elevated propensity to develop cancers compared to the general population. It has been clearly shown that in this population immunosuppression and concomitant infection with oncogenic viruses play an important role. NMSCs are the most frequent cause of cutaneous malignancy in PLWH, and they represent a new oncologic challenge due increasing age of HIV-infected patients. In this paper, we tried to review the incidence of NMSCs among PLWH, any different clinical presentations of squamous cell and basal cell carcinoma between PLWH and HIV negative persons and any differences in efficacy and safety of treatments and response to immunomodulators (see **Table 4**). According to several authors ratios of BCC and SCC are similar between PLWH and HIV negative persons (4:1) (140), with BCC essentially more frequent than SCC. PLWH with NMSCs tend to be younger, to have a higher risk of local recurrence and to have an overall poorer outcome. The main risk factors for NMSCs are similar to HIV negative individuals. Superficial BCC is the most frequent variant and is more often found on the trunk and in multiple lesions. SCC tends to be more aggressive in HIV infected people and it presents at significantly younger age, with higher risk of local recurrence and metastasis (141). The treatment of SCC in people with HIV should be at least aggressive as the treatment in HIV seronegative individuals. There is no strong evidence of how the depth of the immune compromission (CD4 counts) directly influence the risk of NMSCs, and this evidence supports mainly the risk of SCC rather than BCC. It is mandatory to suggest to PLWH a proper screening of all precancerous lesions besides a careful prevention with sun avoidance and use of sunscreen. Notably, there is a lack of official recommendation and guidelines on these subjects. Recently, Vismodegib and Sonidegib, two hedgehog signaling pathway inhibitors, have been approved to treat unresectable BCCs that are not amenable for surgery and radiotherapy. It is difficult to compare the efficacy of Sonidegib and Vismodegib due to the absence of trials designed to prove it and also because the first is only approved for locally advanced BCC while the last is also used for metastasizing BCC (128). It is time to answer to this lack of knowledge with appropriate trials that study the role of targeted therapy for BCC, in PLWH that result to be inoperable. The effectiveness of checkpoint inhibitors targeting the PD-1/PD-L1 pathway in the treatment of many malignancies in PLWH it has been suggested by recent data, hopefully stronger evidence on this matter will follow. In order to improve knowledge, PLWH must be included in immune-oncology studies. In conclusion, the treatment of advanced NMSC represents still an important challenge for clinician, mainly because of the lack of high-quality evidence and randomized trials. Further studies are required to focus on the best therapeutic approaches to NMSCs and mostly on the impact of cancer screening interventions among HIV-infected patients, in order to improve cancer diagnosis at an earlier stage. Further studies are needed to learn and apply pathogenetic insights to obtain new therapeutic options and correlate the degree of HIV-related immunodeficiency with disease outcome.

**Table 4:** Synoptic picture with differences between PLWH and immunocompetent.

	GENERAL POPULATION	HIV POSITIVE PATIENTS
**RATIOS BCC/SCC**	4:1 (30)	4:1 (33)
**MORE FREQUENT SUBTYPE OF BCC**	Nodular (75)	Superficial (137)
**PREVALENCE M/W**	Slight male predilection (after age of 45 years) (138)	Increased risk of NMSC in both male and female patients (139)
**AGE AT DIAGNOSIS**	60 -70 years	Tendency to earlier age (∼ 45 years) (83)
**RISK FACTORS FOR** **DEVELOPMENT OF NMSCs**	Chronic sun-exposure, fair phenotypic features, family history, older age, genetic mutations	The same as for general population plus immunodeficiency and coinfection with oncogenic viruses (83)
**ANATOMIC DISTRIBUTION**	Sun-exposed areas (more frequent on head and neck regions)	Trunk and extremities (generally lesions are multiple) (83)
**CLINICAL COURSE**	Usually indolent; nevertheless, metastatic forms lead to poor patient outcome	Generally more aggressive compared to the general population (83)
**RISK METASTATIC DISEASE**	Generally BCC shows minimal metastatic potential (88); SCC has a 4% annual incidence of metastatic disease (90)	Higher compared to the general population (83)
**TREATMENT**	**Full thickness Treatment** (93)	The same as standard of care for general population. However, it’s mandatory to evaluate the risk of drug interactions (between cART and other treatments) (134)
	Surgical excision	
	Mohs micro-graphic surgery	
	Radiotherapy	
	**Superficial ablative techniques**	
	**Targeted Therapy**	

## Author Contributions

ER, MB, and GN designed the study. ER, MM, MB, and MC performed the screening of articles following the inclusion criteria. ER, MM, and FF wrote the article. CG, MB, and GN supervised the part of diagnosis and treatment. All authors contributed to the article and approved the submitted version.

## Conflict of Interest

The authors declare that the research was conducted in the absence of any commercial or financial relationships that could be construed as a potential conflict of interest.

## Publisher’s Note

All claims expressed in this article are solely those of the authors and do not necessarily represent those of their affiliated organizations, or those of the publisher, the editors and the reviewers. Any product that may be evaluated in this article, or claim that may be made by its manufacturer, is not guaranteed or endorsed by the publisher.
