# Supplementary material for: Non-Melanoma Skin Cancer in People Living With HIV: From Epidemiology to Clinical Management
Source: Front Oncol. 2021 Aug 4;11:689789. doi: 10.3389/fonc.2021.689789 (PMC8371466; doi:10.3389/fonc.2021.689789)
Supplement: Supplementary file 1 [file DataSheet_1.docx]

Full-text articles assessed for eligibility

(n=225)

## Inclusion

Full-text articles excluded, with reasons

(n=15)

(n=11) No original data

(n=1) Not in English, Spanish, Italian

(n=3) No HIV patients

Studies included in qualitative synthesis

(n=210)

Records excluded

(n=2123)

Records after duplicates removed

(n=2445)

Additional records identified on Up to date

(n=5)

Records identified through

Enbase e PubMed

(n=3613)

## Identification

## Screening

Records excluded

(n=97)

## Eligibiliyy

Records screened

(n=322)
